# 

**Published:** 1979

**Authors:** 


### BRISTOL MEDICO-CHIRURGICAL JOURNAL

JULY/OCTOBER 1979
VOLUME 94 (iii/iv)
NUMBER 351/352

